# Comparing Residue Clusters from Thermophilic and Mesophilic Enzymes Reveals Adaptive Mechanisms

**DOI:** 10.1371/journal.pone.0145848

**Published:** 2016-01-07

**Authors:** Deanne W. Sammond, Noah Kastelowitz, Michael E. Himmel, Hang Yin, Michael F. Crowley, Yannick J. Bomble

**Affiliations:** 1 Biosciences Center, National Renewable Energy Laboratory, Golden, Colorado, 80401, United States of America; 2 Department of Chemistry & Biochemistry and the BioFrontiers Institute, University of Colorado, Boulder, Colorado, 80309, United States of America; Weizmann Institute of Science, ISRAEL

## Abstract

Understanding how proteins adapt to function at high temperatures is important for deciphering the energetics that dictate protein stability and folding. While multiple principles important for thermostability have been identified, we lack a unified understanding of how internal protein structural and chemical environment determine qualitative or quantitative impact of evolutionary mutations. In this work we compare equivalent clusters of spatially neighboring residues between paired thermophilic and mesophilic homologues to evaluate adaptations under the selective pressure of high temperature. We find the residue clusters in thermophilic enzymes generally display improved atomic packing compared to mesophilic enzymes, in agreement with previous research. Unlike residue clusters from mesophilic enzymes, however, thermophilic residue clusters do not have significant cavities. In addition, anchor residues found in many clusters are highly conserved with respect to atomic packing between both thermophilic and mesophilic enzymes. Thus the improvements in atomic packing observed in thermophilic homologues are not derived from these anchor residues but from neighboring positions, which may serve to expand optimized protein core regions.

## Introduction

Enzymes have evolved to function in a wide range of conditions, including temperatures up to 130°C [1]. Highly thermostable enzymes are of industrial interest since performing processes at higher temperatures offers benefits such as decreased risk of contamination, increased substrate solubility and higher reaction rates [2]. Understanding adaptive evolutionary response under the selective pressure of high temperature promises to provide a set of rules that can impart desired thermostability to any target protein. In a broader sense, studying thermostable enzymes is important to better comprehend the evolutionary process as well as the energetics that drive protein folding, recognition and stability.

Studies comparing thermostable and mesostable enzymes have identified features associated with enhanced thermostability. Some features seen in thermophilic enzymes contribute to the stability of protein folding, such as the improved quality of packing [3, 4], improved electrostatic interactions [5–7] and increased hydrophobicity in the protein core [8, 9]. Other features diminish destabilizing forces such as decreased conformational flexibility [10–12] or entropy of unfolding [13]. The successes of protein engineering efforts based on these features support they are indeed mechanisms that can enhance thermostability [14, 15]. Often, however, these features are difficult to translate into actionable information that can direct protein-engineering efforts.

Proteins are stabilized by a network of cooperative interactions [16]. Altering one residue alters the local environment for neighboring residues, thus residues that are beneficial in one context can be deleterious in another. Xiao and Honig find electrostatic interactions are more favorable in hyperthermophilic proteins [17]. These ionic interactions are not identified by amino acid composition but instead depend on the location of the ionizable groups. Padgornaia et al. evaluate the limits of natural sequence variation by exhaustively mapping a small, defined region of the PhoQ-PhoP interface, finding functionally active sequence combinations that are not permissible individually [18]. Understanding how interacting residues adapt and evolve to achieve enhanced thermostability is an important step towards capturing beneficial epistatic substitutions. Targeted research aimed at improving our understanding of epistasis could aid the development of new rational protein engineering approaches.

Here we use structural bioinformatics to compare clusters of interacting residues from homologous thermophilic and mesophilic enzymes, allowing the comparison of interacting substitutions in structurally equivalent environments. Our approach is to identify what we call “motifs”, or groups of residues that are adjacent in space and thus interacting, breaking the problem down to smaller context dependent units. By comparing motifs from homologous thermophilic and mesophilic enzymes we can better see sequence evolution within the local chemical environment. Using carefully matched and characterized proteins from different enzyme families we can observe specific trends within or across families. We place emphasis on cellulose enzymes as the absence of certain cellulase family members in thermophilic organisms raises the question of whether some protein folds are not well suited for thermostability [19]. Further, by comparing structurally equivalent clusters we are able to evaluate backbone alterations resulting from the evolutionarily selected mutations.

## Results

We compare paired thermostable and mesostable homologues to investigate changes in local environment for groups of interacting residues. We select homologous enzymes covering structurally and catalytically distinct families (Table 1). Each family of enzymes contains a thermophilic or hyperthermophilic member as well as less thermostable homologues. The Carbohydrate-Active EnZymes database organizes enzymes into families based on structural similarities (CAZy, www.cazy.org) [20]. We select industrially relevant glycoside hydrolase (GH) families for investigation given the high interest research focused on the global need for alternative liquid fuel sources [21]. We add additional enzyme families from the published literature [8, 22–24].

**Table 1:** Characteristics of the enzyme families selected for cluster analysis, with the most thermostable member highlighted in bold.

PDB	ORGANISM	T_OPT_ (°C)	% Sequence ID	RMSD	MW (kDa)
**GH5 (β/α)**_**8**_	** **	** **	** **	** **	** **
**3amc**	***Thermotoga maritima***	**80**	**100**	**0.00**	**36.5**
3jug	*Bacillus sp*. *N16-5*	70	17	2.45	32.7
1gzj	*Thermoascus aurantiacus*	70	15	2.11	33.6
2whj	*Bacillus agaradhaerens*	60	17	2.40	34.2
**GH7 (β-jelly roll)**					
**4csi**	***Humicola grisea* var. *thermoidea***	72.5***	**100**	**0.00**	**46.8**
1cel	*Trichoderma reesei*	62.5***	58	0.56	45.9
2yg1	*Heterobasidion irregulare *	45	61	0.50	46.9
**GH9 (α/α)**_**6**_					
**4dod**	***Caldicellulosiruptor bescii***	~75*	**100**	**0.00**	**51.0**
1ks8	*Nasutitermes takasagoensis*	67	43	0.62	47.8
3wc3	*Eisenia fetida*	40	56	0.54	48.5
**GH10 (β/α)**_**8**_	* *				
**1vbr**	***Thermotoga maritima***	**90**	**100**	**0.00**	**38.2**
2uwf	*Bacillus halodurans*	70	30	0.90	41.0
2f8q	*Bacillus sp*. *NG-27*	70	31	0.97	40.8
1hiz	*Bacillus stearothermophilus*	65	29	0.86	43.4
1b30	*Penicillium simplicissimum*	65	33	0.79	32.4
1k6a	*Thermoascus aurantiacus*	63	33	0.68	32.8
1n82	*Bacillus stearothermophilus*	60	33	0.89	38.5
**GH11 (β-jelly roll)**					
1f5j**	***Dictyoglomus thermophilum***	**75**	**100**	**0.00**	**22.3**
3zse**	***Thermobifida fusca***	**75**	**(100)**	**(0.00)**	**20.8**
1m4w	*Thermopolyspora flexuosa*	70	48 (82)	0.70 (0.40)	21.8
2nqy	*Bacillus agaradhaerens*	70	56 (52)	0.51 (0.58)	22.7
1igo	*Bacillus subtilis B230*	60	58 (52)	0.71 (0.64)	22.5
1hix	*Streptomyces sp*. *S38*	60	47 (76)	0.82 (0.61)	20.1
1bk1	*Aspergillus kawachii*	60	38 (41)	0.87 (0.69)	19.7
3m4f	*Scytalidium acidophilum*	50	37 (42)	0.97 (0.71)	19.3
1enx	*Trichoderma reesei II*	45	51 (56)	1.09 (0.81)	20.7
1xnd	*Trichoderma harzianum*	45	51 (55)	0.73 (0.57)	20.7
1xnb	*Bacillus circulans*	45	43 (62)	0.72 (0.54)	20.4
1ukr	*Aspergillus niger*	40	38 (40)	0.91 (0.71)	19.7
1xyn	*Trichoderma reesei I*	40	38 (41)	0.71 (0.59)	19.0
**GH13 (β/α)**_**8**_	* *				
**1ciu**	***Thermoanaerobacterium thermosulfurigenes***	**60**	**100**	**0.00**	**75.4**
1cyg	*Bacillus stearothermophilus*	55	68	0.51	75.4
1cdg	*Bacillus circulans*	35	70	0.49	74.5
**Lactate dehydrogenase (βαβ)**	* *				
**1a5z**	***Thermatoga maritima***	**85**	**100**	**0.00**	**34.2**
1ldn	*Bacillus stearothermophilus*	55	41	1.17	34.7
5ldh	pig	37	34	2.03	35.8
6ldh	dogfish	15	37	1.33	36.3
**Malate dehydrogenase (βαβ)**	* *				
**1bmd**	***Thermus flavus***	**75**	**100**	**0.00**	**35.3**
4mdh	pig	37	54	0.77	36.3
1emd	*Escherichia coli*	37	21	3.16	32.4
**Methionine aminopeptidase (pita-bread fold)**					
**1xgs**	***Pyrococcus furiosus***	**90**	**100**	**0.00**	**32.8**
1mat	*Escherichia coli*	-	24	1.24	29.2
1y1n	*Mycobacterium tuberculosis*	50	20	1.13	30.6

* The published activity temperature for *C*. *bescii* GH9 (PDB 4dod) includes a CBM3 domain.

** GH11 includes two thermophilic enzymes with the same optimum activity temperature. % Sequence ID and RMSD compared against 3zse.pdb are shown in parentheses.

*** The stability presented is T_m_ (°C), not optimum activity temperature.

Optimum organism growth temperature is a good indicator of enzyme activity temperature. However, the conformational flexibility needed for a given mechanism can also be a strong determinant of optimal activity temperature [25]. The questions investigated here rely on the accurate assignment of relative thermostability of homologous enzymes. Therefore, selected enzymes have experimentally determined structures and experimentally determined optimum activity temperatures. Further, additional protein domains can significantly alter the stability of a given muti-domain enzyme [26, 27]. For this work we select enzymes with a single catalytic domain or where an optimum activity temperature has been determined for the catalytic domain alone. We make one exception and include the industrially relevant GH9 from *C*. *bescii*, for which a CBM3 was present in the activity temperature measurements [28].

### Identifying structurally equivalent clusters of interacting residues

**Fig 1:**
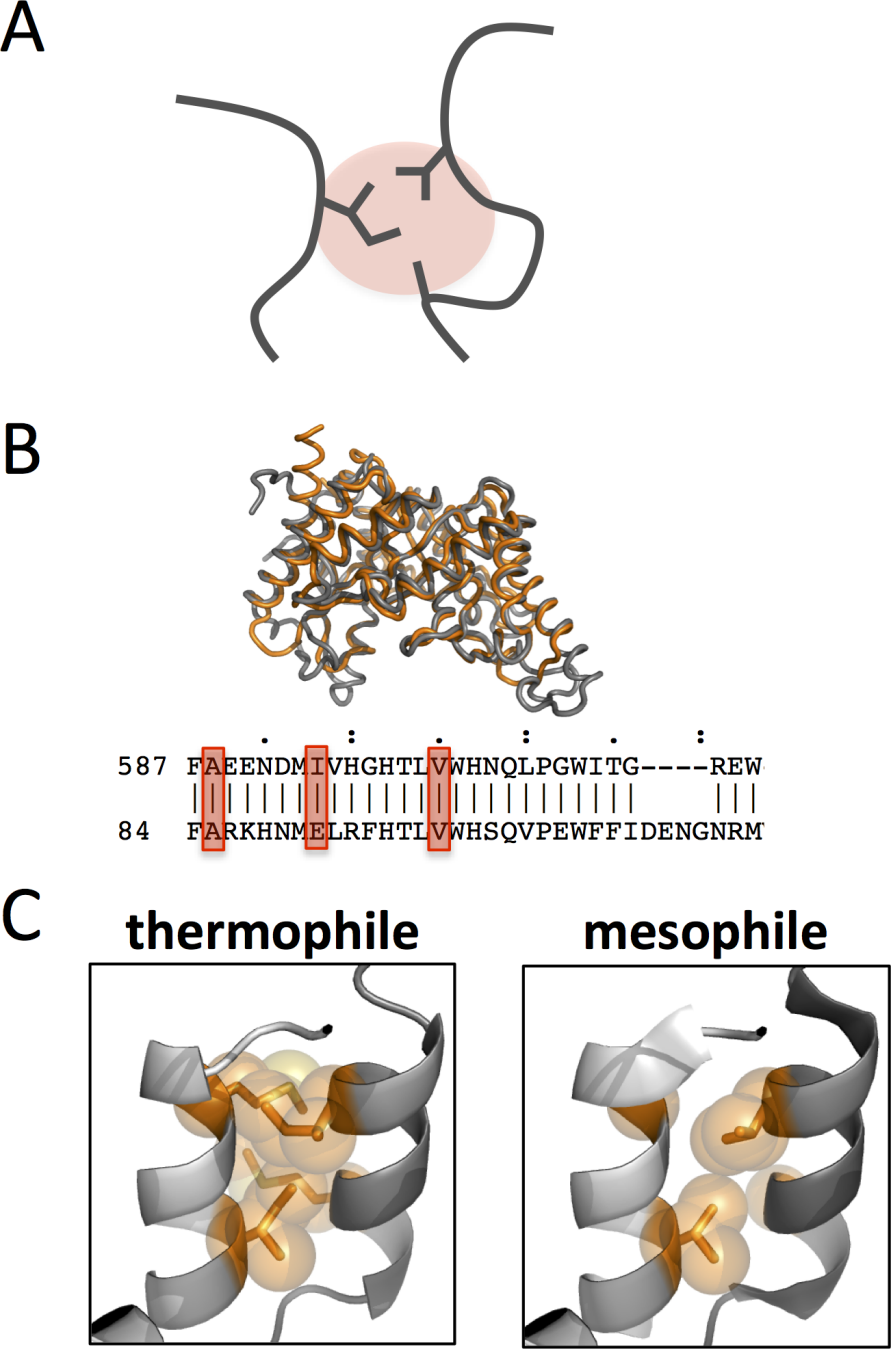
Identifying equivalent clusters in homologous proteins allows for direct comparison of local environments. (A) A cartoon depiction of cluster of adjacent residues is shown (red circle). (B) Structural alignment of paired enzymes is shown, with PDB 1vbr in orange and 2uwf in gray. The structurally aligned residues for the paired enzymes are shown beneath. (C) Differences in atomic packing is depicted with alternate sequences shown in stick and sphere representation on PDB 2wva.

Residues that are close in space, or interacting, can display energetic cooperativity [29–31]. Here, clusters of spatially adjacent residues are identified using a distance cutoff. Two residues are defined as interacting if any side chain heavy atoms (C, N, O and S) are within 3 Å (Fig 1A). A structure is tiled in clusters, starting with the N-terminal residue and identifying adjacent positions and ultimately moving to the C-terminal residue.

Equivalent clusters in homologous enzymes are identified using the structural alignment algorithm, jFatCat flexible [32]. jFatCat flexible allows for a minimum number of backbone rotations to maximize the identification of structurally similar regions. The flexible structural alignment also generates an alignment of structurally matched sequence positions (Fig 1B). Thus clusters from paired proteins can be accurately identified and compared based on local context (Fig 1C).

### Residue clusters display a high degree of sequence variation between thermophilic and mesophilic enzymes

Residue substitutions can display epistasis, and the effects of multiple substitutions cannot be easily predicted from knowledge of the individual substitutions. Podgornaia and Laub, mapping the sequence space of the PhoQ-PhoP interface, found combinations of mutations producing functionally fit proteins in cases where the individual substitutions resulted in loss of function [18]. These results suggest evolutionary constraints and limitations of directed evolution. However, comparing homologous proteins from distantly related organisms, as is included here, can be used to investigate adaptive mechanisms taking place over long evolutionary timescales and encompassing large sampling of allowed sequence space.

**Fig 2:**
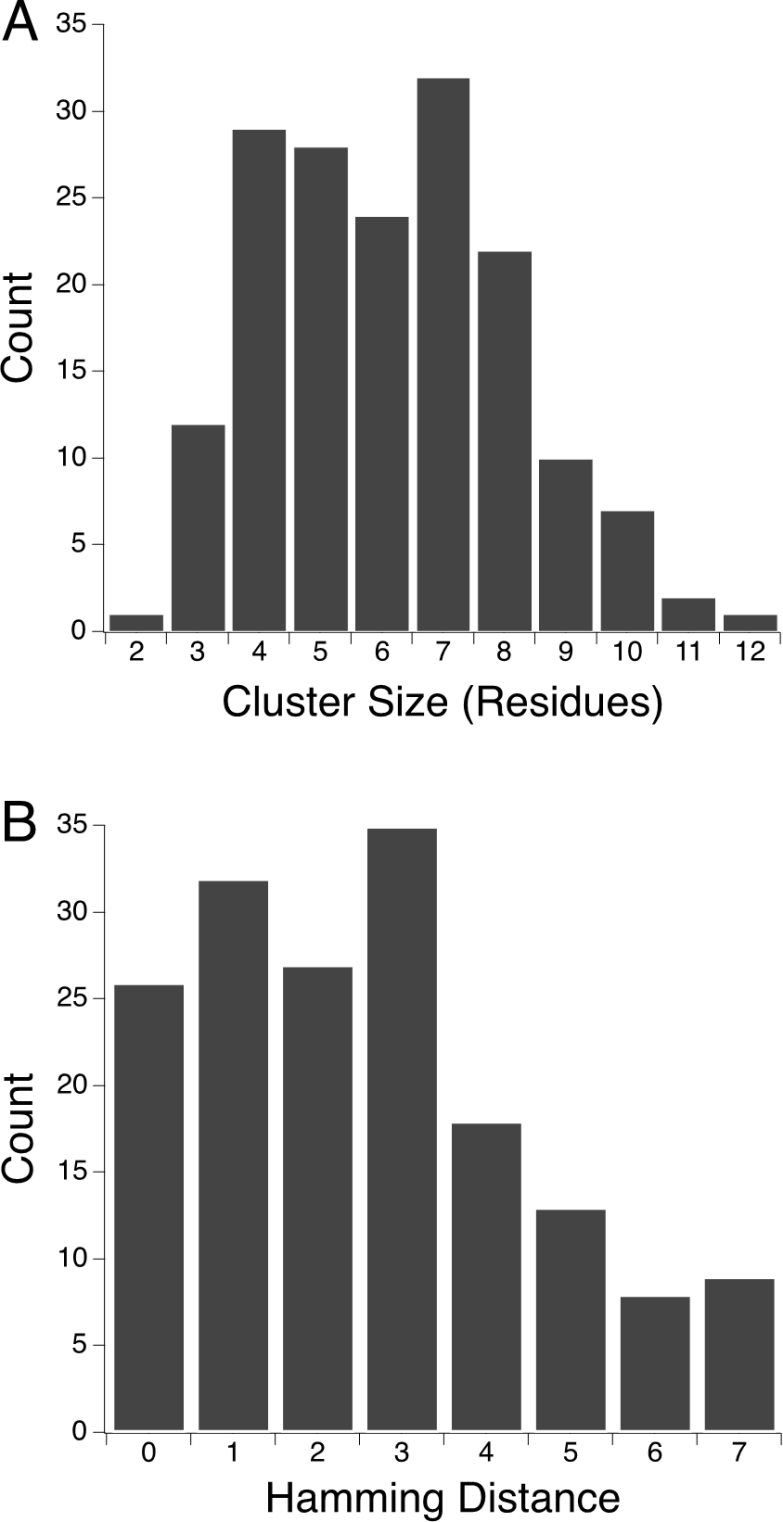
Evaluating the potential for epistasis. (A) The number of residues in each motif are determined for all representative thermophilic-mesophilic structure pairs and binned according to the motif size. (B) The number of residue substitutions, given as Hamming distance, in each equivalent thermophilic-mesophilic motif is determined and binned.

Protein families in our dataset have from three to thirteen member structures (Table 1). We take a representative protein pair from each family to avoid biasing the results towards enzyme families with more members. Cluster sizes and number of amino acid substitutions are taken from pairs of structures representing the most and least thermostable members in each family. We evaluate the average size of the motifs by determining number of residues in each cluster (Fig 2A). We also evaluate the average number of amino acid substitutions, expressed as Hamming distance, between the paired clusters (Fig 2B). One hundred and fifty five motifs are identified in the representative dataset. Motifs range from two to twelve residues in size, with eighty percent of motifs having four to eight residues.

The total sequence identities between paired homologous thermophilic and mesophilic enzymes in our dataset range from fifteen to eighty two percent (Table 1). Residues in the protein core are generally more conserved compared to total sequence identity, as protein function requires properly folded structure [33, 34]. Indeed, thirty five percent of the equivalent thermophilic-meosophilic enzyme clusters from the representative dataset have zero or one residue substitution. Despite the overall high conservation within hydrophobic protein core regions, sixty five percent of the motifs have a Hamming distance equal to or greater than two, with up to seven substitutions observed. Given the clusters are composed of interacting residues, these results highlight the potential for cooperative interactions and the need for a better understanding of epistatic effects.

### Residue clusters are optimized for atomic packing in thermophilic enzymes

Improved quality of side-chain packing, or absence of cavities, is often observed in more thermostable enzymes relative to mesophilic homologues [35, 36]. Here equivalent clusters are evaluated for quality of packing using the solvent accessible surface area with the van der Waals radii expanded by 1.4 Å to represent a water molecule (SASA_1.4_) [37]. The SASA_1.4_ is determined for all residues in a motif, comparing thermophilic enzyme clusters to equivalent clusters in paired mesophilic enzymes (ΔSASA_1.4_). Thus, a negative ΔSASA_1.4_ indicates the thermophilic enzyme cluster displays fewer or smaller cavities and thus improved atomic packing compared to the mesophilic enzyme cluster.

**Fig 3:**
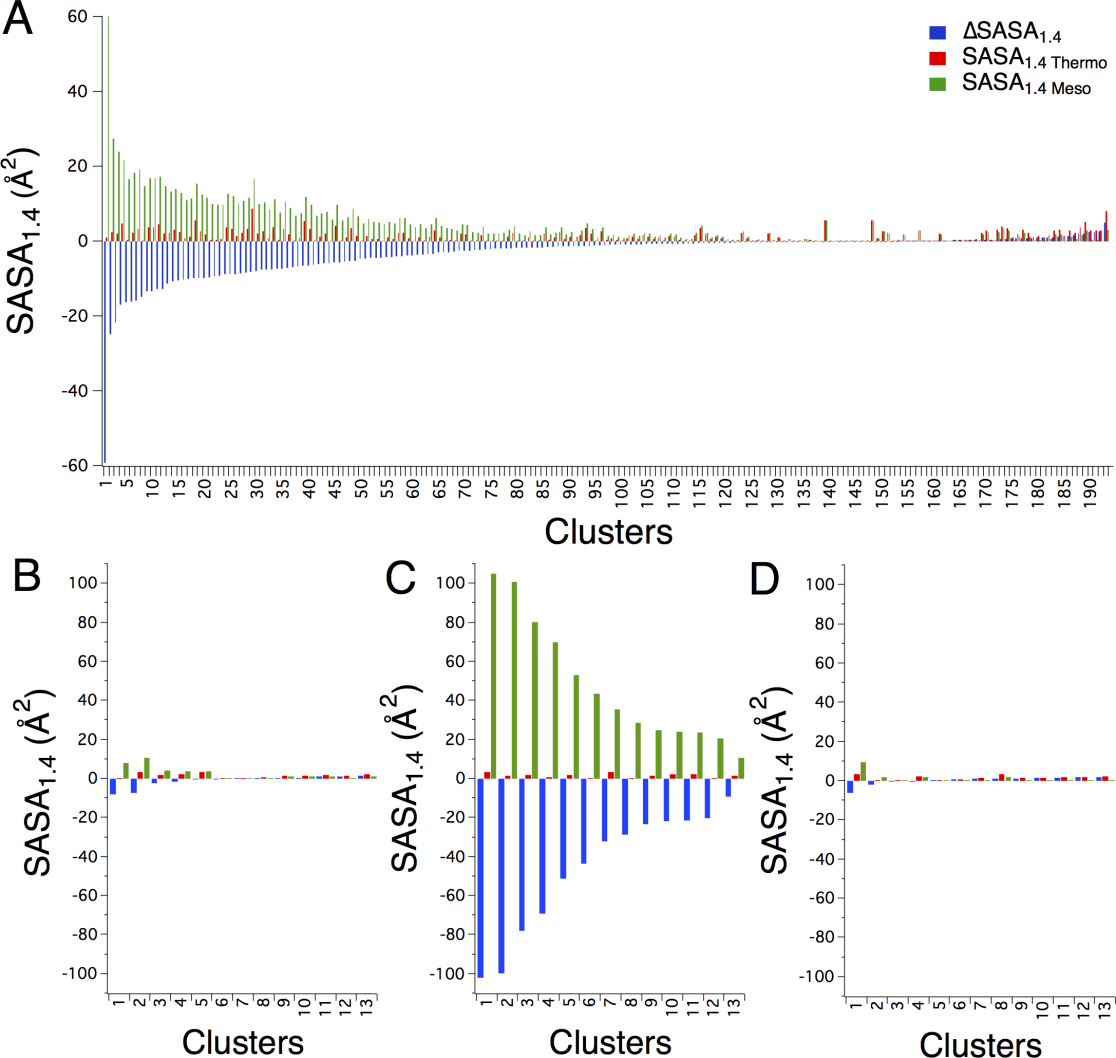
Thermophilic enzyme clusters display closer atomic packing compared to mesophilic enzyme clusters for most enzyme pairs evaluated. (A) SASA_1.4_ values for clusters from the representative thermophilic-mesophilic structure pairs are shown, with thermophilic clusters shown in red, mesophilic clusters in green and the difference, ΔSASA_1.4_, in blue. Values are sorted by ΔSASA_1.4_. (B) SASA_1.4_ values are shown comparing clusters from the thermophilic (PDB 1a5z) and mesophilic (PDB 6ldh) lactate dehydrogenase enzymes, which have a difference in optimum activity temperature of 30°C. (C) the thermophilic (PDB 1a5z) and mesophilic (PDB 5ldh) lactate dehydrogenase enzymes, with a difference in optimum activity temperature of 48°C, (D) and the thermophilic (PDB 1a5z) and psychrophilic (PDB 1ldh) lactate dehydrogenase enzymes, with a difference in optimum activity temperature of 70°C.

Again, representative thermophile-mesophile enzyme pairs with the most and least thermostable enzymes are used to prevent bias towards enzyme families with the largest representation. The majority of clusters in thermophilic enzymes display improved atomic packing relative to mesophilic enzymes, with seventy-two percent of the clusters having a negative ΔSASA_1.4_ (Fig 3A and Table 2). Proteins are dynamic molecules, often exhibiting discrete conformational substates. In this work, however, we are assessing single protein conformations. As a result ΔSASA_1.4_ values less than but close to zero could simply be the result of comparing single conformations from experimentally determined structures. We therefore consider ΔSASA_1.4_ values less than or greater than 3 Å. Of the representative structures, thirty-three percent of the clusters from thermophilic enzymes exhibit improved atomic packing of greater than 3 Å while only one percent of clusters from mesophilic enzymes exhibit superior atomic packing of greater than 3 Å (Table 2).

**Table 2:** Comparing void volumes, as determined by ΔSASA_1.4_ and residue contact number, and percent sequence identity for paired clusters.

**Representative Structures—193 clusters**		
	**Count**	**Percentage**
**ΔSASA**_**1.4**_ **< 0**	145	75%
**ΔSASA**_**1.4**_ **< 1**	64	33%
**ΔSASA**_**1.4**_ **< 2**	1	1%
	**Max**	**Average**
**SASA**_**Thermo**_	8.7	1.5
**SASA**_**Meso**_	60.2	4.8
**All Structures—501 clusters**		
	**Count**	**Percentage**
**ΔSASA**_**1.4**_ **< 0**	388	77%
**ΔSASA**_**1.4**_ **≤ -3**	227	45%
**ΔSASA**_**1.4**_ **≥ 3**	6	1%
**ΔContact Number ≤ 0**	346	69%
	**Max**	**Average**
**SASA**_**Thermo**_	8.7	1.6
**SASA**_**Meso**_	100.9	8.4
** **		
**% Sequence Identity**		
	**Median**	**Average**
**ΔSASA**_**1.4**_ **≤ -3**	50	49
**-3 < ΔSASA1.4 < 3**	67	65
**ΔSASA**_**1.4**_ **≥ 3**	44	51

ΔSASA_1.4_ analysis comparing equivalent clusters in thermophilic and mesophilic enzymes generally identifies the more thermostable enzyme of a homologous pair. This approach does not, however, predict the rank-order for a family of homologous enzymes based on thermal stability. ΔSASA_1.4_ analysis was performed comparing the most thermostable enzyme in each family to every other family member. ΔSASA_1.4_ for the entire dataset shows the same trend seen with the representative structures, although some structure pairs exhibit larger differences in ΔSASA_1.4_ (Fig 3A, S1 Fig and Table 2). The maximum SASA_1.4_ observed for a mesophilic enzyme motif in the representative protein set is 60.2 Å^2^, while the maximum SASA_1.4_ observed for a mesophilic enzyme motif in the entire dataset is 100.9 Å^2^ (Table 2). The representative dataset compares the most and least thermostable enzymes from each family, yet larger differences in SASA_1.4_ can be found in paired enzymes with closer optimum activity temperatures.

To verify our findings we evaluate our entire dataset with an alternative method used to measure the quality of atomic packing in protein structures. We use the VLDP web server (http://www.dsimb.inserm.fr/dsimb_tools/vldp/) to compute residue contact number for each residue cluster. VLDP uses a Laguerre Tessellation to evaluate residue volumes in protein structures.[38] The results similar to SASA_1.4_, show thermophilic residue clusters are more likely to have a higher residue contact number compared to the equivalent mesophilic residue cluster (Table 2).

Further, while only smaller void volumes are observed in thermophilic residue clusters from every family evaluated here, improved atomic packing between equivalent thermophilic and mesophilic clusters is not observed for all pairs of homologous proteins. For example, differences in atomic packing between a thermophilic lactate dehydrogenase (PDB 1a5z) and two homologous mesophilic enzymes and one psychrophilic enzyme are strikingly different. Both a mesophilic lactate dehydrogenase (PDB 1ldn) and a psychrophilic lactate dehydrogenase (PDB 6ldh) show small void volumes, very similar to the SASA_1.4_ seen in the thermophilic lactate dehydrogenase (Fig 3B and 3D). Yet the differences in optimum activity temperatures compared to the thermophilic homologues are 30° and 70°C respectively. Comparing the same thermophilic lactate dehydrogenase to a different mesophilic homologue (PDB 5ldh) yields a ΔSASA_1.4_ distribution commonly seen in the protein pairs evaluated here, with significant enhancement see in atomic packing of the thermophilic residues (Fig 3C).

Similarly, the GH13 structures (PDB 1ciu) and (PDB 1cdg), with a 25°C difference in optimum activity temperatures, display ΔSASA_1.4_ values close to zero for all equivalent residue clusters (S2 Fig). The GH13 residue clusters also display high sequence identity, with the average percent sequence identity of 92, and 21 of the 33 clusters with one hundred percent sequence identity. These results indicate that while mesophilic and psychrophilic enzymes can have larger void volumes, other mechanisms also contribute to the lower thermal stability. However, the thermophilic enzymes do not appear to tolerate the destabilizing larger void volumes seen in many paired mesophilic enzymes investigated here.

### Tiling clusters of interacting residues uncovers conserved “anchor” positions

The algorithm is designed to identify buried clusters of neighboring, interacting residues by searching for neighboring residues starting with N-terminal residues and cycling to C-terminal residues. A protein structure is thus tiled in partially overlapping clusters. As a result, some buried residues with many neighbors are found in multiple clusters. These residues, described here as the anchor residues, resemble what are termed hot-spot residues when found at protein-protein interfaces. Hot spot residues at protein-protein interfaces are positions that contribute a significant amount of the stabilizing energy to drive the interaction.

**Fig 4:**
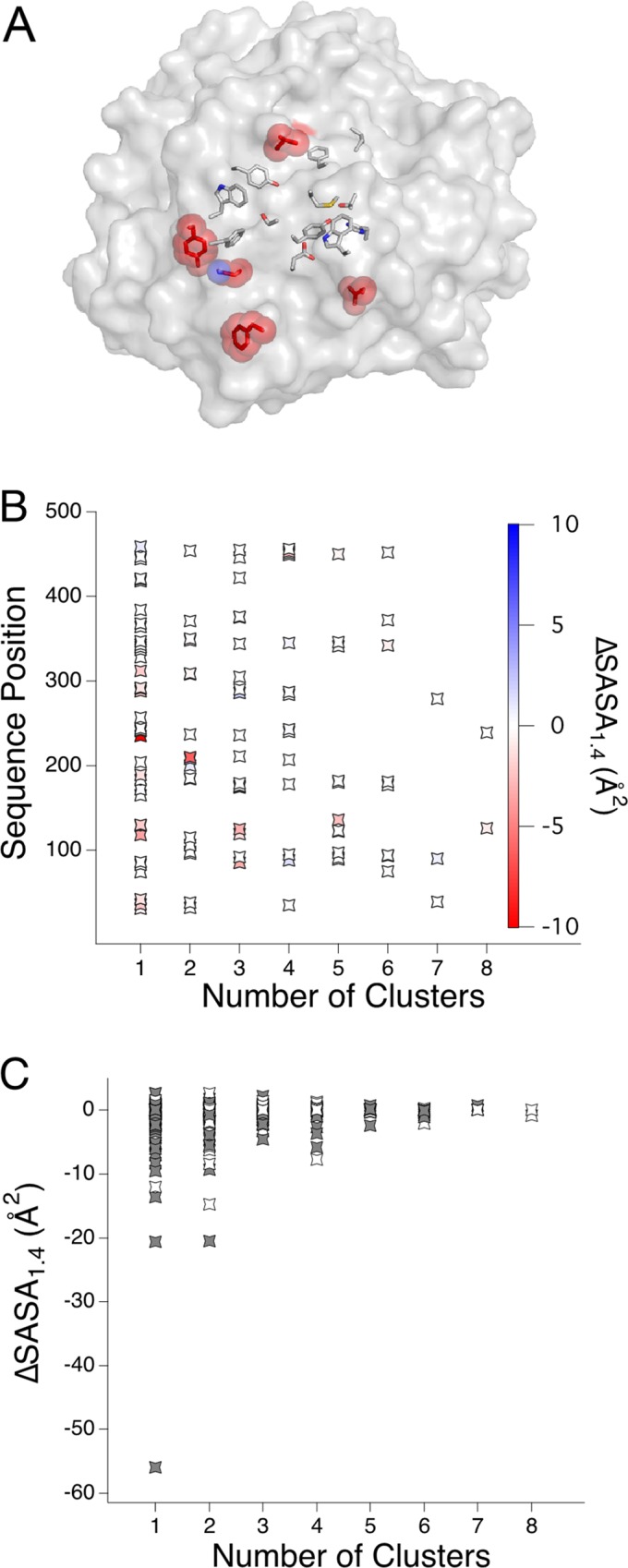
Anchor residues are conserved in atomic packing. (A) The thermostable GH9 (PDB 4dod) is shown in surface representation, with anchor residues that are seen in a larger number of clusters shown in stick representation. Residues exhibiting the largest ΔSASA_1.4_, which are never anchor residues, are colored red. (B) Sequence positions from 4dod are binned by the number of clusters in which they are found. The heat scale indicates ΔSASA_1.4_. Importantly, blue is not observed as there are no mesophilic clusters with significantly better atomic packing relative to the matched thermophilic cluster. (C) Sequence positions from the representative set of structures are binned by the number of motifs in which they are found (x-axis), with ΔSASA_1.4_ shown for each paired position (y-axis). A white symbol indicates sequence conservation, and gray indicates the sequence differs at that position.

Interestingly, SASA_1.4_ is conserved for the anchor residues found here. For example, in Fig 4A, residues for the thermostable GH9 structure (PDB 4dod) found in six or more motifs are shown in stick representation, while the residues exhibiting the largest ΔSASA_1.4_ compared to equivalent residues in mesophile counterpart (PDB 3wc3) are highlighted in red. All residues in the GH9 thermophile are plotted according to the number of motifs in which they are found. Each sequence position is colored according to a heat map, where blue indicates the ΔSASA_1.4_ is positive, with better packing in the mesophilic motif, and red indicates negative ΔSASA_1.4_ with better packing in the thermophilic motif. The scale is set to ±10 ΔSASA_1.4_, as the lowest ΔSASA_1.4_ value for this pair of structures is -11. Blue is not observed for any sequence position (Fig 4B).

Evaluating this trend for sequence positions found in the representative structure pairs shows that in fact ΔSASA_1.4_ is conserved for all anchor residues, defined as positions found in five or more motifs (Fig 4C). The same trend holds for all sequence positions in the entire dataset, again for all anchor residues found in five or more motifs (S3 Fig). Thus the large improvements in ΔSASA_1.4_ found in thermophilic clusters compared to mesophilic clusters come from residues making fewer contacts rather than the anchor regions of the protein core. Each sequence position is colored white if the sequence identity is conserved and grey if identity is not conserved (Fig 4C). Interestingly, despite the conservation in ΔSASA_1.4_, the sequence identities for these anchor residues are not absolutely conserved. However, a higher degree of sequence conservation is seen in residue clusters with similar atomic packing, as determined by ΔSASA_1.4_ between -3 and 3 Å^2^ (Table 2). In fact, 82% of residue clusters with 100% sequence identity are found in the residue clusters that also display conserved atomic packing.

### Backbone adjustments as determined by distance differences

**Fig 5:**
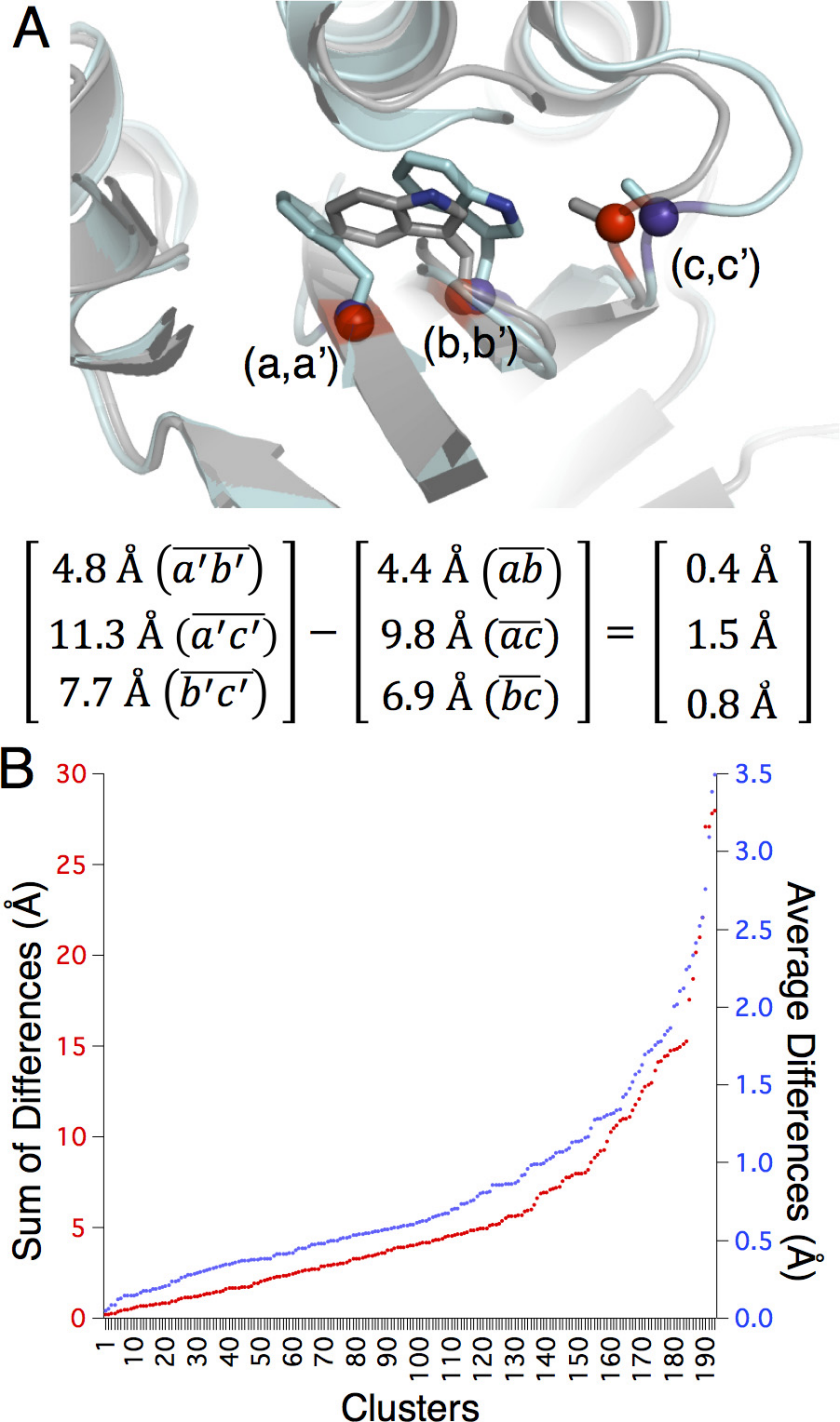
The backbone can move significantly in the structurally equivalent clusters. (A) Three Cα atoms from a paired cluster are shown in red spheres (thermophilic enzyme) and purple spheres (mesophilic enzyme). The atoms are labeled a, b and c for the thermophilic enzyme and a’, b’ and c’ for the mesophilic enzyme. The Euclidian distances between Cα atoms are shown for each enzyme, with the distance differences at right. (B) The sum of the absolute values for the distance differences (red), and the average distance differences (blue) for each representative cluster are shown, sorted by summed or averaged distances.

The networks of interacting side-chain clusters identified here tend to be large and contain multiple amino acid substitutions. Understanding how the protein backbone responds to accommodate alternate sequence combinations helps pinpoint the challenges for molecular design algorithms. Distance difference matrices are ideal for the comparison of geometric and distance similarities in enzymes and enzyme active sites as structural alignments are not necessary. Comparing distance matrices between structurally equivalent clusters is similar to comparing enzyme active sites. Distances between all Cα atoms are determined for each paired thermophilic and mesophilic cluster (Fig 5A).

Taking the absolute value of each Cα distance difference allows the differences to be summed. In this way a single metric measures the degree of backbone movement between paired clusters. Further, dividing each sum by the number of residues normalizes the summed differences, resulting in a comparable metric regardless of cluster size. The resulting metric shows that, on one hand, approximately half of the clusters from the representative dataset display little backbone movement, yielding an average summed distance difference of zero to approximately one half Å (Fig 5B). The distance differences increase rapidly, however, for the remainder of motifs. Over twenty-five Å sum-of-distances is observed, and an average distance difference of up to three and a half Å. Equivalent residue clusters with an average distance difference of 3 Å have an average of 3 Å between each pair of Cα residues. Residue clusters displaying high average distance differences likely have different shapes or sizes. Thus, while some paired motifs exhibit structural conservation, many display significant backbone movement. These results highlight the complexities of predicting potentially epistatic groupings of residues, even in a relatively small and defined protein region.

## Discussion

A key challenge in protein engineering is accurately modeling energetic changes from mutations close in space. Protein double mutant free energy cycles show that non-additivity is a common phenomenon, especially when residue pairs are close in space [29–31]. Evolutionary studies also indicate sequence alterations can be cooperative [39, 40]. Evaluating sequence changes is more informative in context of the internal structural and chemical environment in which the substitutions are found. Ultimately, accurately predicting epistatic effects from multiple mutations requires a better understanding of how local environment affects amino acid substitutions. Here we examine how proteins evolve to function at high temperatures by evaluating local regions, applying structural informatics to investigate evolutionary patterns.

Optimized atomic-packing in protein core regions is important for enhanced thermostability. Cavities found in protein core regions diminish thermostability [36], and void volumes sized to accommodate water molecules have been observed more often in psychrophilic enzymes compared to homologous mesophilic counterparts [35]. Poorly packed protein core regions can lead to loss of conformational stability [41, 42]. Cavity filling mutations, conversely, can increase the hydrophobicity of the protein core [36]. As a protein engineering approach, Chen et al. demonstrated decreasing cavities in a protein hydrophobic core could enhance thermostability by transposing hydrophobic core regions from three thermostable enzymes into a mesophilic homologue [4]. Here, interacting residues in thermophilic enzymes display optimized van der Waals interactions, as seen by minimized cavities, compared to their mesophilic counterparts.

The results also support the role of alternative mechanisms leading to large changes in enzyme thermal stability. In addition to the examples discussed above (Fig 3B and 3D), Arimori et al., comparing two GH 9 enzymes with a 27°C difference in optimum activity temperature (1ks8 and 3nc3 in Table 1), identify an excess of negatively charged amino acids on the surface as the destabilizing mechanism for the psychrophilic homologue [43]. Kalimeri et al. report similar findings, comparing thermophilic and mesophilic malate dehydrogenase orthologues. They find that atomic volume is the same for both othologues, and instead oligomerization leads to enhanced thermal stability [44]. Importantly, regardless of the imperfections contributing to the moderate stability of mesophilic enzymes with ideal atomic packing, residue clusters from thermophilic enzymes appear to always display ideal atomic packing. Thus, as a designable element, these residue clusters represent evolutionarily optimized motifs.

The term hot-spot residues describes key positions that contribute a majority of the binding energy to protein-protein interfaces [45]. Similar to hot-spot residues, the approach applied here uncovers anchor residues that make many contacts and are thus found in many clusters. These anchor residues are conserved with regards to atomic packing in both thermophilic and mesophilic homologues. The observed improvements in atomic packing for thermophilic enzymes are thus found in residues that are peripheral to core anchor residues. These peripheral sequence positions might, therefore, serve to expand optimized protein core regions such as the residue clusters encompasing anchor residues.

The anchor residues, while well conserved in atomic packing, are not absolutely conserved in sequence. Putting other energetic contributions aside, the absence of cavities, which are known to be energetically deleterious, appears to be important for thermostability. Further, energetic contributions may be met without having to hold key sequence positions to absolute conservation. These results also support the importance of considering buried residues in structural context, as SASA for a given residue is not determined simply by that residue but by that residue and it’s neighbors.

While anchor residues are not absolutely conserved in sequence, higher sequence conservation is seen in the residue clusters that display similar atomic packing between thermophilic and mesophilic enzymes. Conversely, residue clusters that differ in atomic packing also show higher divergence in sequence. Since optimized atomic packing is seen disproportionately in the thermophilic homologues, the results indicate these protein regions have evolved to confer additional stabilization in the thermophilic homologues.

Obtaining the desired physicochemical properties for some protein targets may not always be achievable by combinations of single sequence substitutions. Yet evaluating all possible sequence space even in a small, defined region results in a combinatorial explosion that renders the approach intractable. The challenge, based on measured conformational changes, is backbone movement often seen when comparing equivalent residue clusters. Modeling, or predicting, such conformational changes with no *a priori* knowledge of optimized target sequences is not trivial and explains the challenges in predicting beneficial sequence combination *in silico*.

The method presented here identifies evolutionary optimized residue clusters with ideal sequence combinations and side-chain packing patterns. Importantly, these results suggest that while mesophilic and psychrophilic enzymes can accommodate cavities in the protein core, thermophilic enzymes cannot. As such, all residue clusters from the core of thermophilic enzymes can be viewed as potential transposable motifs to evaluate successful sequence combinations on complementary backbone structures.

## Experimental Procedures

### Protein Dataset

Thermophilic and mesophilic glycoside hydrolase enzymes were identified from the CAZy database (Carbohydrate-Active enZYmes), which categorizes enzymes based on structural similarity [20]. Enzymes were evaluated for the presence of additional domains using Pfam [46]. Enzyme optimum activity temperatures were found in the following publications: GH5 [26], GH 7 [47, 48], GH 9 [43, 49], GH10, GH 11, GH13 and lactate dehydrogenase and malate dehydrogenase [23], and methionine aminopeptidase [50, 51].

Sequences were aligned and analyzed using the MacVector software (MacVector, Inc., Cary, NC) [52]. Sequence alignments were performed using the GONNET substitution matrix [53], with a gap opening penalty of 10 and a gap extension penalty of 0.05. The molecular weight for each protein was computed based on the amino acid sequence using the ExPASy ProtParam tool [54]. Root-mean-square deviations (RMSD) for paired thermophilic and mesophilic enzymes were computed using PyMol (The PyMOL Molecular Graphics System, Version 1.5.0.4 Schrödinger, LLC.)

### Identification of Residue Clusters

Interacting residue clusters were identified using a distance cutoff of 3 Å between side chain heavy atoms (C, N, O and S) using the protein design software, Rosetta [55, 56]. Structurally equivalent residue clusters in homologous mesophilic enzymes were identified using the structural alignment algorithm, jFatCat flexible [32]. Residue clusters were filtered based on degree of solvent accessibility, selecting only clusters where each residue displayed less than 3 Å^2^ of SASA as determined using Naccess [57]. Residue clusters were excluded if an equivalent residue from a thermophilic cluster was not found in the mesophilic cluster.

Distance difference matrices were calculated for paired thermophilic and mesophilic clusters. Accurate structural alignment of paired residues was verified manually if the sum of the distance differences for a cluster exceeded 20 Å.

### Comparing Structurally Equivalent Residue Clusters

The residue accessible surface areas were computed using the program Naccess [57]. Naccess rolls a probe of a given radius over the van der Waals surface of a molecule to trace the accessible surface. A probe of radius 1.4 Å was used here to reflect the radius of water and thus the solvent accessible surface area.

Histograms for cluster size and Hamming distance were created using StatPlus:mac [58]. Graphs were generated using IGOR Pro (WaveMetrics Inc., Lake Oswego, OR).

## Supporting Information

S1 Fig SASA_1.4_ values for clusters from all remaining thermophilic-mesophilic structure pairs not shown in Fig 3A are shown, with thermophilic clusters shown in red, mesophilic clusters in green and the difference, ΔSASA_1.4_, in blue. Clusters are sorted by ΔSASA_1.4_.
(DOCX)

S2 Fig SASA_1.4_ values are shown comparing clusters from the thermophilic (PDB 1ciu) and mesophilic (PDB 1cdg) GH13 structures, which have a difference in optimum activity temperature of 25° C yet small differences in SASA_1.4_ between clusters. (DOCX)

S3 Fig Sequence positions from all paired structures are binned by the number of motifs in which they are found (x-axis), with ΔSASA_1.4_ shown for each paired position (y-axis). A white symbol indicates sequence conservation, and gray indicates the sequence differs at that position.
(DOCX)
