# Supplementary material for: Comparing Residue Clusters from Thermophilic and Mesophilic Enzymes Reveals Adaptive Mechanisms
Source: PLoS One. 2016 Jan 7;11(1):e0145848. doi: 10.1371/journal.pone.0145848 (PMC4704809; doi:10.1371/journal.pone.0145848)
Supplement: S1 Fig — Clusters are sorted by ΔSASA1.4. (DOCX) [file pone.0145848.s001.docx]

**S1 Fig.** SASA_1.4_ values for clusters from all remaining thermophilic-mesophilic structure pairs not shown in Figure 3A are shown, with thermophilic clusters shown in red, mesophilic clusters in green and the difference, ΔSASA_1.4_, in blue. Clusters are sorted by ΔSASA_1.4_.
