# Supplementary material for: Comparing Residue Clusters from Thermophilic and Mesophilic Enzymes Reveals Adaptive Mechanisms
Source: PLoS One. 2016 Jan 7;11(1):e0145848. doi: 10.1371/journal.pone.0145848 (PMC4704809; doi:10.1371/journal.pone.0145848)
Supplement: S2 Fig — (DOCX) [file pone.0145848.s002.docx]

**S2 Fig.** SASA_1.4_ values are shown comparing clusters from the thermophilic (PDB 1ciu) and mesophilic (PDB 1cdg) GH13 structures, which have a difference in optimum activity temperature of 25° C yet small differences in SASA_1.4_ between clusters.
