# Supplementary material for: Comparing Residue Clusters from Thermophilic and Mesophilic Enzymes Reveals Adaptive Mechanisms
Source: PLoS One. 2016 Jan 7;11(1):e0145848. doi: 10.1371/journal.pone.0145848 (PMC4704809; doi:10.1371/journal.pone.0145848)
Supplement: S3 Fig — A white symbol indicates sequence conservation, and gray indicates the sequence differs at that position. (DOCX) [file pone.0145848.s003.docx]

**S3 Fig.** Sequence positions from all paired structures are binned by the number of motifs in which they are found (x-axis), with ΔSASA_1.4_ shown for each paired position (y-axis). A white symbol indicates sequence conservation, and gray indicates the sequence differs at that position.
